# Understanding the facilitators and barriers to integrating trachoma interventions into routine health systems

**Published:** 2023-01-30

**Authors:** Sarity Dodson, Alemu Gemechu, Yeneneh Mulugeta Deneke, Mitiku Bekere, Shambel Belete

**Affiliations:** Research Director: The Fred Hollows Foundation, Melbourne, Australia.; Programme Manager: The Fred Hollows Foundation, Addis Ababa, Ethiopia.; Medical advisor for Africa and the Middle East: The Fred Hollows Foundation, Addis Ababa, Ethiopia.; TT-surgery Transitioning Project Coordinator: The Fred Hollows Foundation, Addis Ababa, Ethiopia.; NTD Technical Advisor: Federal Ministry of Health, Addis Ababa, Ethiopia

## Abstract

Identifying context-specific barriers to integration is essential to achieve the elimination of trachoma as a public health problem.

Achieving vision for all requires eye care services and health systems that are equipped to manage the full spectrum of eye conditions affecting populations. However, in many settings, interventions for trachoma, the world's leading infectious cause of blindness, are delivered outside of countries’ routine health care system. In part, this is due to trachoma's classification as one of the neglected tropical diseases (NTDs) that are addressed via national NTD programmes. In other situations, this is necessary due to the lack of eye care services at primary health care level that is available to the communities affected by trachoma.

There is wide recognition that sustaining the elimination of trachoma as a public health problem will require moving trachoma interventions out of NTD programmes and integrating them into routine public health services and sustainable health systems. One reason for this is so that incident cases of trachomatous trichiasis (TT) can be managed by national eye health services in a post-elimination setting.

Moving trachoma interventions into existing health systems is complicated by numerous factors which are highly context specific and must be understood in order to effectively transition the services. In 2021, the Ethiopian Federal Ministry of Health, in collaboration with The Fred Hollows Foundation and the Oromia Regional Health Bureau, began implementation research to understand how best to do this. Although Ethiopia has been proactive in developing trachoma transition guidelines, the complexity of this work warranted further research to understand:

- the detail of the activities involved across all levels of the health system
- the costs
- the mode of care
- the barriers and enablers to transitioning.

**Figure:**
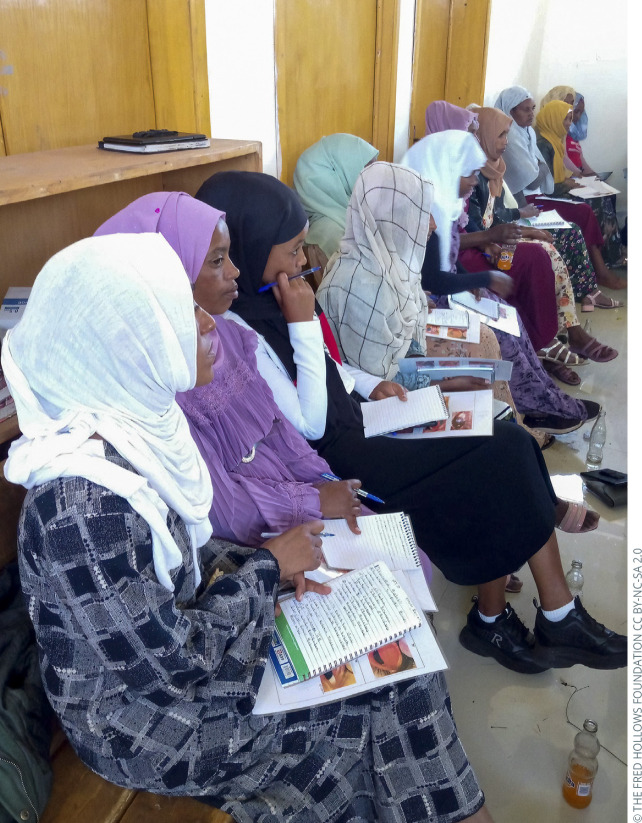
Health extension workers in Sude district training for the transition of trachomatous trichiasis case referral. ethiopia

So far, four key areas have been identified as affecting the successful transition of trachoma interventions into Ethiopia's national health system.

## 1. Health system capacity

Capacity assessments have identified many primary health care facilities that experience human resource constraints, including a lack of trained personnel to deliver trachoma interventions. Many of these health facilities also lack the infrastructure and medical supplies to effectively manage patients with TT.

## 2. Supply chain challenges

Ongoing global economic challenges also have a local impact and have resulted in a reduced number of suppliers as well as significant delays in the delivery of medical products, including materials for TT operations. 

## 3. Persistent and recrudescent trachoma

This study selected two *woredas* (districts) due to their low prevalence of trachoma (less than the elimination threshold). However, recent surveillance surveys found that, in one of the two *woredas*, the prevalence of trachoma had increased to above the World Health Organization (WHO) threshold for trachoma elimination. Persistent and recrudescent trachoma have several implications for transition planning. Most notably, settings above the WHO elimination threshold must receive trachoma interventions, including surgery and antibiotics, through community outreach rather than facility-based care. 

## 4. Community awareness

A knowledge, attitudes, and practices (KAP) survey conducted in the study area highlighted low levels of community awareness about trachoma. Inadequate understanding of trachoma in the community affects transition by reducing service uptake by the affected community members.  

In response to some of these challenges, the Oromia Regional Health Bureau, in collaboration with The Fred Hollows Foundation, is strengthening the capacity of primary health centres to provide trachoma interventions, including TT surgery. This includes training integrated eye care workers to be able to provide  TT surgery, and training Health Extension Workers (HEWs) to identify trachoma and TT, so that people suspected to have either could be referred to the nearest health centre. HEWs are also being provided with a counselling guide in local languages so that they can effectively communicate about trachoma to affected communities and raise awareness about the disease, thereby improving the uptake of interventions.

Going forward, the lessons learnt from this project will be critical to informing national transitioning efforts in Ethiopia and overcoming barriers to successful transition. Investment in human resources and community sensitisation will help to overcome some of these challenges. Other challenges, including persistent and renewed outbreaks of trachoma, and external challenges such as consistent and reliable supply chains, threaten the successful transition of services. 

For Ethiopia to eliminate trachoma as a public health problem and to achieve universal eye health coverage, a clear plan is needed to respond to incident cases and prevent a resurgence of the disease. Ethiopia's National Transitioning Guidelines, and other resources, including the International Coalition for Trachoma Control's transition toolkits, provide important support for this process. Continued structured research and effective knowledge exchange is needed to support NTD programmes.

We hope that sharing these experiences with eye health professionals across all trachoma endemic countries will help to drive global progress and navigate these complex trachoma end-game challenges.

